# BET Protein Inhibitor JQ1 Ameliorates Experimental Peritoneal Damage by Inhibition of Inflammation and Oxidative Stress

**DOI:** 10.3390/antiox12122055

**Published:** 2023-11-29

**Authors:** Vanessa Marchant, Flavia Trionfetti, Lucia Tejedor-Santamaria, Sandra Rayego-Mateos, Dante Rotili, Giulio Bontempi, Alessandro Domenici, Paolo Menè, Antonello Mai, Catalina Martín-Cleary, Alberto Ortiz, Adrian M. Ramos, Raffaele Strippoli, Marta Ruiz-Ortega

**Affiliations:** 1 Cellular and Molecular Biology in Renal and Vascular Pathology Laboratory, IIS-Fundación Jiménez Díaz, School of Medicine, Universidad Autónoma de Madrid, 28040 Madrid, Spain; vanessa.marchant@quironsalud.es (V.M.); lucia.tejedor@quironsalud.es (L.T.-S.); srayego@fjd.es (S.R.-M.); 2 RICORS2040, 28029 Madrid, Spain; aortiz@fjd.es (A.O.); amramos@fjd.es (A.M.R.); 3 Gene Expression Laboratory, National Institute for Infectious Diseases, Lazzaro Spallanzani IRCCS, 00149 Rome, Italy; flavia.trionfetti@inmi.it (F.T.); giulio.bontempi@uniroma1.it (G.B.); raffaele.strippoli@uniroma1.it (R.S.); 4 Department of Molecular Medicine, Sapienza University of Rome, 00161 Rome, Italy; 5 Department of Drug Chemistry and Technologies, Sapienza University of Rome, 00185 Rome, Italy; dante.rotili@uniroma1.it (D.R.); antonello.mai@uniroma1.it (A.M.); 6 Renal Unit, Department of Clinical and Molecular Medicine, Sant’Andrea University Hospital, Sapienza University of Rome, 00189 Rome, Italy; alessandro.domenici@uniroma1.it (A.D.); paolo.mene@uniroma1.it (P.M.); 7 Laboratory of Nephrology, IIS-Fundación Jiménez Díaz, School of Medicine, Universidad Autónoma de Madrid, 28040 Madrid, Spain; cmartinc@fjd.es

**Keywords:** BET proteins, JQ1, peritoneal damage, inflammation, oxidation, NRF2

## Abstract

Peritoneal dialysis (PD) is a current replacement therapy for end-stage kidney diseases (ESKDs). However, long-term exposure to PD fluids may lead to damage of the peritoneal membrane (PM) through mechanisms involving the activation of the inflammatory response and mesothelial-to-mesenchymal transition (MMT), leading to filtration failure. Peritoneal damage depends on a complex interaction among external stimuli, intrinsic properties of the PM, and subsequent activities of the local innate–adaptive immune system. Epigenetic drugs targeting bromodomain and extra-terminal domain (BET) proteins have shown beneficial effects on different experimental preclinical diseases, mainly by inhibiting proliferative and inflammatory responses. However the effect of BET inhibition on peritoneal damage has not been studied. To this aim, we have evaluated the effects of treatment with the BET inhibitor JQ1 in a mouse model of peritoneal damage induced by chlorhexidine gluconate (CHX). We found that JQ1 ameliorated the CHX-induced PM thickness and inflammatory cell infiltration. Moreover, JQ1 decreased gene overexpression of proinflammatory and profibrotic markers, together with an inhibition of the nuclear factor-κB (NF-κB) pathway. Additionally, JQ1 blocked the activation of nuclear factor erythroid 2-related factor 2 (NRF2) and restored changes in the mRNA expression levels of NADPH oxidases (NOX1 and NOX4) and NRF2/target antioxidant response genes. To corroborate the in vivo findings, we evaluated the effects of the BET inhibitor JQ1 on PD patients’ effluent-derived primary mesothelial cells and on the MeT-5A cell line. JQ1 inhibited tumor necrosis factor-α (TNF-α)-induced proinflammatory gene upregulation and restored MMT phenotype changes, together with the downmodulation of oxidative stress. Taken together, these results suggest that BET inhibitors may be a potential therapeutic option to ameliorate peritoneal damage.

## 1. Introduction

The prevalence of chronic kidney disease (CKD) is increasing year by year, and it is estimated to become the fifth leading cause of death at the end of this century [1]. A relevant number of CKD patients progress to end-stage kidney disease (ESKD) and require kidney replacement therapy (KRT) in substitution for the vital detoxification function of the kidneys [2]. Peritoneal dialysis (PD) is a KRT technique that takes advantage of the natural permeability properties of the peritoneal membrane to interchange waste products and fluids between the blood and the PD solution placed in the peritoneal cavity. PD has advantages for patients, namely fewer dietary restrictions, more tolerability of side effects, and, above all, it is an at-home modality that allows patients to increase their quality of life. However, long-term PD may cause chronic peritoneal damage; this, combined with recurrent peritonitis episodes, leads to ultrafiltration failure and therapy discontinuation [3]. There is currently no effective therapy to prevent or delay this pathological process.

The long-lasting exposure of the peritoneum to PD fluids, which contain proinflammatory metabolites, such as glucose and lactate, induces a sustained inflammatory response, characterized by the activation of mesothelial cells (MCs) and overproduction of cytokines and chemokines, leading to the recruitment of leukocytes in the peritoneal cavity. The sustained inflammatory response also contributes to the induction of phenotypic changes in MCs, named the mesothelial-to-mesenchymal transition (MMT), resulting in cell migration and extracellular matrix (ECM) accumulation in the submesothelial zone [4,5]. In this pathological scenario, neovascularization, chronic inflammation, and fibrosis occur, leading to a dramatic reduction in the peritoneal ultrafiltration ability and the discontinuation of PD [3].

Recent studies have reported that epigenetic modifications are involved in the damage of the peritoneal membrane (PM), including DNA methylation, post-translational histone modification, and noncoding RNAs. These epigenetic mechanisms modify the activities of signaling molecules, transcriptional factors, and gene transcription, therefore contributing to the promotion of peritoneal damage [6]. In this sense, the pharmacological or genetic inhibition of histone methyltransferase activities has been found to ameliorate fibrosis in experimental mouse models of fibrosis induced by methylglyoxal (MGO) or chlorhexidine gluconate (CHX) [7,8]. Accordingly, histone acetyltransferase (HAT) or histone deacetylase (HDAC) inhibitors restrain the MMT phenotype in MCs and the in vivo fibrotic outcome [9,10,11].

The family of bromodomain and extra terminal (BET) proteins, that includes bromodomain-containing protein (BRD)2, BRD3, BRD4, and BRDT, is a class of epigenetic transcriptional modulators. These proteins, through their tandem *N*-terminal bromodomains (BD1 and BD2), bind to acetylated lysine residues on histones (and other nuclear proteins) and transcription factors, playing a key role in chromatin-based cellular processes, including gene transcription and chromatin remodeling [12]. BRD4, the most studied BET protein member, may regulate transcription at several levels. This protein has an intrinsic kinase activity that stimulates RNA polymerase II (RNA pol II)-dependent transcription [13]. BRD4 also stimulates transcription by binding to acetylated residues in the promoter or enhancer regions of proinflammatory genes and transcription factors, including nuclear factor-κB (NF-κB) [14]. Many preclinical studies have demonstrated that the pharmacological inhibition of BET proteins exerts protective effects on proliferative, inflammatory, and fibrotic pathologies [12,15,16]. Initiatives using small-molecule BET inhibitors, such as JQ1 and I-BET, have been explored in clinical trials searching for antiproliferative antitumor therapies and inflammatory disorders [17]. However, no studies have been reported investigating whether BET inhibition could target peritoneal damage. Toward this objective, we have evaluated the effect of BET inhibitor JQ1 on preclinical peritoneal damage induced by the intraperitoneal administration of CHX in mice, as well as in cultured MCs, investigating the potential molecular mechanisms that may be involved.

## 2. Materials and Methods

### 2.1. Ethics Statement

All the animal procedures were performed on C57BL/6 male mice according to the guidelines of animal research in the European Community and with prior approval by the Animal Ethics Committee of the Health Research Institute IIS-Fundación Jiménez Díaz (PROEX 242.2/21). Experiments on effluent-derived MCs from ESKD patients in PD replacement therapy were performed according to guidelines from the ethics committee of Sant’Andrea Hospital, Sapienza University (Rome, Italy). Written informed consent was obtained from all the PD patients. The protocol and informed consent were reviewed and approved by the Ethics Committee of Clinical Investigation at Sapienza University, ref: 4697_2017 (Roma, Italy).

### 2.2. Chlorhexidine Gluconate-Induced Peritoneal Damage in Mice

Studies were performed on adult male C57BL/6 mice (9–12 week-old mice obtained from Charles Rivers, Barcelona, Spain) maintained at local animal facilities, with free access to food and water and normal light/dark cycles and under special pathogen-free conditions. Peritoneal damage was induced in mice by intraperitoneal injections of 0.1% chlorhexidine gluconate (CHX) dissolved in saline solution (0.9% NaCl), as previously described by other authors [18], with a minor modification. CHX was delivered daily at a volume of 10 mL/kg of mouse weight for 10 consecutive days. At the same time, a group of CHX-treated mice (*n* = 8) was intraperitoneally injected with JQ1 (50 mg/kg of weight) [19] dissolved in 10% hydroxypropyl β-cyclodextrin in sterile water every day until the end of the model, using DMSO as the vehicle. JQ1, a thieno-triazolo-1,4-diazepine, was synthesized at the Department of Drug Chemistry and Technologies at Sapienza University of Rome (Mai laboratory). CHX-treated mice without the JQ1 treatment formed the CHX group (*n =* 6). Mice without treatment were used as the control group (*n =* 4). Mice were euthanized, and parietal peritoneal tissue samples were collected according to the specific requirements for the subsequent analyses.

### 2.3. Cell Culture

The human mesothelial cell line MeT-5A (ATCC, Rockville, MD, USA) was cultured in Earle’s M199 medium supplemented with 10% FBS (Gibco, Billings, MT, USA), 2 mM L-glutamine (EuroClone, Milano, Italy), 100 U/mL penicillin, and 100 μg/mL streptomycin (Gibco, Billings, MT, USA).

Human-effluent-derived primary MCs were isolated from four PD patients, as previously described [20]. Written informed consent was obtained from all the PD patients. The protocol and informed consent were reviewed and approved by the Ethics Committee of Clinical Investigation at Sapienza University, ref: 4697_2017 (Roma, Italy). The demographic and clinical features of these patients are summarized in Supplementary Table S1. Effluent-derived MCs were cultured in Earle’s M199 medium supplemented with 10% FBS (Gibco, Billings, MT, USA), 2 mM L-glutamine (EuroClone, Milano, Italy), 100 U/mL penicillin, 100 μg/mL streptomycin (Gibco, Billings, MT, USA), and amphotericin B (2.5 μg/mL).

Both the MeT-5A cell line and primary MCs were grown at 37 °C in a humidified atmosphere with 5% CO_2_. The cells were pretreated with 1 μM JQ1 for 3 h (when applicable), followed by 24 h of stimulation with TNF-α (5 ng/mL) as a proinflammatory stimulus or with TGF-β1 (2 ng/mL) combined with IL-1β (5 ng/mL) as a fibrosis/MMT-inducing stimulus.

### 2.4. Histological and Immunohistochemical Analyses

Peritoneal tissue sections were collected from the mice, fixed in 4% formaldehyde, embedded in paraffin, and cut in 3–4 μm tissue sections for immunohistochemistry (IHC) studies and Masson’s trichrome staining (Bio-Optica, Milano, Italy), following the manufacturer’s instructions. For IHC analysis, the tissue sections were deparaffined and hydrated, and antigen retrieval was carried out using a sodium citrate buffer (10 mM, pH 6 or 9, according to the particular antigen to be detected) on a PT Link system (DAKO, Santa Clara, CA, USA). An endogenous peroxidase blockade was conducted using 3% hydrogen peroxide (Millipore, MA, USA), and a protein blockade was conducted using Casein blocking solution (Vector Laboratories, Newark, CA, USA). Then, primary antibodies were incubated overnight at 4 °C, followed by incubation with biotinylated secondary antibodies (anti-rabbit or anti-rat, 1:200) and the avidin–biotin complex (ABC; Vector Laboratories, CA, USA). Signals were detected using the 3,3-diaminobenzidine (DAB) chromogen and substrate solution (Abcam, Cambridge, UK). Finally, slides were counterstained with hematoxylin (Merck, Darmstadt, Germany), dehydrated, and mounted with DPX (Merck, Darmstadt, Germany). The following primary antibodies were used: rabbit anti-CD3 (1:100; A0452, DAKO, CA, USA), rat anti-F4/80 (1:50; MCA497, Bio-Rad, Hercules, CA, USA), and rabbit anti-phospho(S40)-NRF2 (1:2000; AB76026, Abcam). Microscopy imaging was performed with a Nikon Eclipse E400 microscope connected to a Nikon digital camera DXM1200F, using ACT-1 software, version 2.63 (Nikon Corporation, Tokio, Japan). The peritoneal membrane (PM) thickness was determined in the Masson staining photomicrographs by measuring the submesothelial zone using the ImageJ tool version 1.53e (five measurements/field), and IHC quantification was conducted by counting positive staining cells in 5–10 randomly chosen fields (200× magnification).

### 2.5. Protein Level Studies

The total protein from frozen peritoneal tissue and cultured cells was isolated by homogenization in T-PER lysis buffer (Thermo Scientific, Waltham, MA, USA) with a 10 μL/mL protease inhibitor cocktail, 0.2 mmol/L PMSF, and 0.2 mmol/L orthovanadate. Proteins were quantified using a Pierce BCA protein assay kit (Thermo Scientific, MA, USA) and then separated by electrophoresis using 8–10% polyacrylamide-SDS gels under reducing conditions for western blotting. Samples were then transferred onto polyvinylidene difluoride membranes (Thermo Scientific, MA, USA), blocked with 5% non-fat milk, and incubated overnight at 4 °C with the following primary antibodies: rabbit anti-phospho(S536)-NF-κB p65 (1:1000; #3031, Cell Signaling, Danvers, MA, USA), mouse anti-phospho(123)-IκBα (1:1000; sc-8404, Santa Cruz, CA, USA), rabbit anti-Fibronectin (1:5000; AB2033, Millipore, Burlington, MA, USA), and rabbit anti-phospho(S465/467)-SMAD2/3 (1:1000; D27F4, Cell Signaling; MA, USA). Then, the membranes were incubated with HRP-conjugated secondary antibodies (anti-rabbit or anti-mouse, 1:5000). Loading controls were done using a mouse anti-GAPDH antibody (1:5000; CB1001, Millipore, Burlington, MA, USA). Proteins on membranes were visualized using a chemiluminescence detection method (Immobilon Crescendo Western HRP substrate, Millipore, Burlington, MA, USA) on an Amersham Imager 600 instrument (GE Healthcare, Arlington Heights, IL, USA). Images were analyzed using densitometry and the ImageJ 1.53e image-processing program bundled with Java 1.8.0_72 (64-bit). The densitometric values of each band were corrected using GAPDH band values and represented as n-folds with respect to the control. For the cell culture experiments, protein level values were expressed as the mean ± SEM of three independent experiments (*n* = 3 for each experimental condition). For mouse sample immunoblotting, protein level values were expressed as the mean ± SEM of 4–8 mice per group.

### 2.6. Gene Expression Assays

The total RNA was isolated from cells and peritoneal tissue by homogenization with TRItidy G (PanReac, Darmstadt, Germany). Next, cDNA was synthesized using a high-capacity cDNA reverse-transcription kit (Applied Biosystems, Waltham, MA, USA), starting from 2 µg of total RNA. Genetic expression analysis was determined by quantitative PCR using the commercial master mix Premix Ex Taq (Takara, Kyoto, Japan) and predesigned TaqMan-based qPCR assays (Supplementary Table S2) containing FAM- or VIC-labeled probes for target and housekeeping genes, respectively, and run on the 7500 Fast Real-Time PCR System thermocycler (Applied Biosystems, CA, USA). Relative expression values were normalized using a housekeeping genetic expression (*Gapdh* and *GAPDH* for mouse and human genes, respectively) and the 2^−ΔΔCt^ method. The results were expressed as fold changes (n-folds) relative to the control.

### 2.7. Statistical Analysis

All the results are expressed as the mean ± SEM. In most of the figures, the results are represented as the n-fold increase with respect to the control. The Shapiro–Wilk test was used to evaluate the sample normality distribution. For samples following the Gaussian normal distribution, one-way ANOVA followed by paired or unpaired *t* tests were performed to compare between the groups. To compare the non-Gaussian samples, the non-parametric Mann–Whitney U test was used. The statistical analysis was conducted using GraphPad Prism 8.0 (GraphPad Software, San Diego, CA, USA). Values of *p* < 0.05 were considered as statistically significant.

## 3. Results

### 3.1. BET Inhibition Ameliorated Experimental Peritoneal Damage in Mice

**Figure 1:**
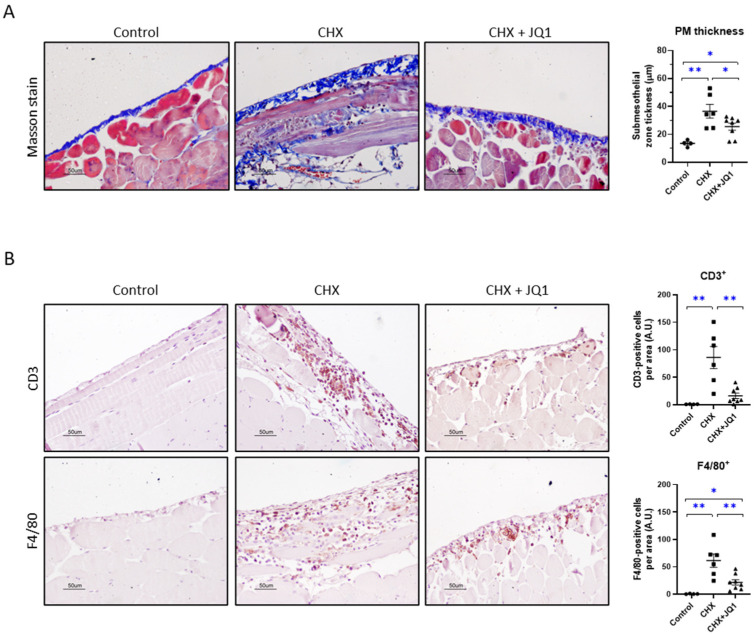
The JQ1 treatment diminished peritoneal membrane (PM) thickness and inflammatory cell infiltration in chlorhexidine gluconate (CHX)-treated mice. Peritoneal damage was induced in C57Bl/6 mice by the daily administration of 0.1% CHX for 10 days. CHX-treated mice were treated daily with JQ1 (50 mg/kg/day) or the vehicle. Histological analyses were conducted on 3–4 μm formalin-fixed paraffin-embedded sections of parietal peritoneum tissues. (**A**) PM thickness was determined by measuring submesothelial zone (blue staining) in Masson’s trichrome staining images. (**B**) Immunohistochemistry used to detect tissue-infiltrated inflammatory cells with specific antibodies (CD3^+^ lymphocytes and F4/80^+^ monocyte/macrophages). Microscopy images correspond to a representative animal from each group (200× magnification). Scale bar: 50 μm. Results are expressed as the mean ± SEM of 4–8 animals per group. * *p* < 0.05; ** *p* < 0.01 according to *t* test.

Chlorhexidine gluconate (CHX) administration is commonly used to experimentally induce peritoneal fibrosis in mice and study different mechanisms of damage [21]. This model is characterized by robust peritoneal fibrosis and chronic inflammation after 3–4 weeks of treatment [22,23,24], although mild fibrosis and inflammation have been reported with shorter CHX treatments (1–2 weeks) [18,25]. In this study, we induced peritoneal damage in mice by the daily intraperitoneal delivery of 0.1% CHX for 10 consecutive days. To evaluate the effect of BET inhibition on this peritoneal damage model, a group of CHX-treated mice was also treated with the BET inhibitor JQ1 (50 mg/kg/day). We found that after 10 days of treatment, CHX induced PM fibrosis, as noted by submesothelial zone thickening characterized by the collagen deposition observed in Masson’s staining images (Figure 1A).

Treatment with JQ1 ameliorated the PM thickness in response to CHX exposure (Figure 1A). Importantly, the CHX-injured peritoneum also presented inflammatory cell infiltration of CD3^+^ lymphocytes and F4/80^+^ macrophages, which was significantly diminished by the JQ1 treatment (Figure 1B). Taken together, these results show that BET inhibition by JQ1 reduces peritoneal damage in a 10-day CHX mouse model.

### 3.2. BET Inhibition Improved CHX-Induced Peritoneal Fibrosis in Mice

**Figure 2:**
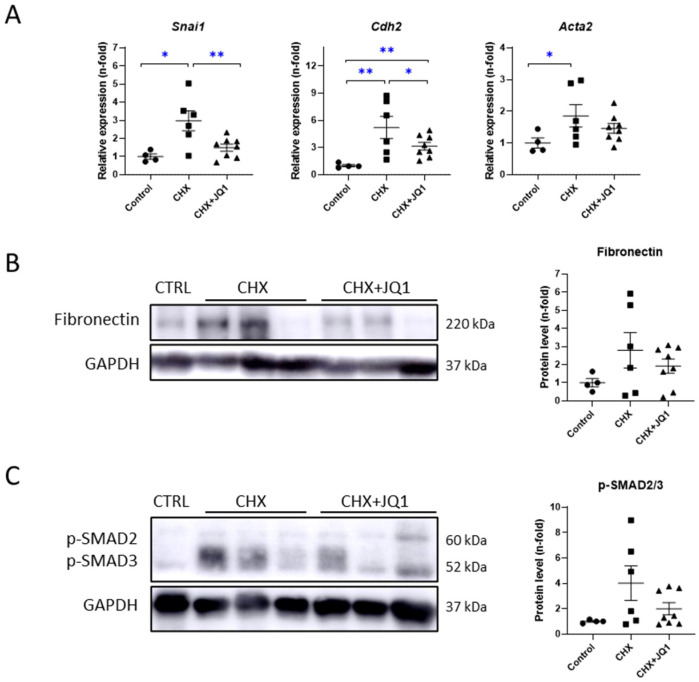
JQ1 diminished CHX-induced changes in mesothelial-to-mesenchymal (MMT) and fibrotic markers in mouse peritoneum. (**A**) Relative expression of MMT-related genes was assayed using RT-qPCR and calculated using the 2^−ΔΔCt^ method. Fibronectin (**B**) and p-SMAD2/3 (**C**) protein levels were evaluated using western blot and quantified using densitometric measurements. Results are represented as n-folds with respect to control group and are expressed as the mean ± SEM of 4–8 animals per group. * *p* < 0.05; ** *p* < 0.01 according to *t* test.

As mentioned before, established peritoneal fibrosis occurs in mice after longer CHX treatments, where the MMT process contributes to PM damage development through signaling pathways as the transforming growth factor-β/suppressor of mothers against decapentaplegic (TGF-β/SMAD). In this study, we found that CHX treatment for 10 days induced a significant increase in the gene expression of MMT/fibrosis-related factors, such as Snail (*Snai1*), N-cadherin (*Cdh2*), and α-smooth muscle actin (*Acta2*); while the JQ1 treatment reduced their expression levels (Figure 2A). Regarding protein levels, CHX-treated mice showed increased fibronectin (Figure 2B) and p-SMAD2/3 (Figure 2C) levels in the peritoneum, and JQ treatment tended to decrease these values. Thus, BET inhibition decreased the expression of MMT and fibrosis factors in a mouse model of peritoneal damage.

### 3.3. BET Inhibition Decreased CHX-Induced Peritoneal Inflammation in Mice

**Figure 3:**
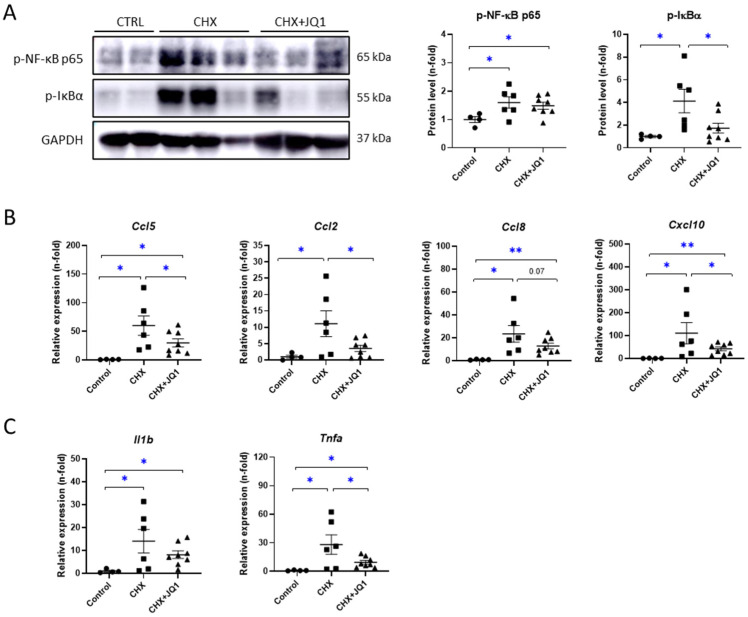
JQ1 inhibited CHX-induced NF-κB pathway activation and gene overexpression of inflammatory markers in mouse peritoneum. (**A**) Peritoneal levels of p-NF-κB p65 and p-IκBα were evaluated using western blotting of total protein from parietal peritoneum and were quantified using densitometric measurements. Left: representative immunoblot images. Right: immunoblot quantification. Relative expression levels of genes encoding (**B**) chemokines (*Ccl5*, *Ccl2*, *Ccl8*, and *Cxcl10*) and (**C**) cytokines IL-1β and TNF-α (*Il1b* and *Tnfa* genes, respectively) were evaluated using RT-qPCR and calculated using the 2^−ΔΔCt^ method. Results are represented as n-folds with respect to control group and are expressed as the mean ± SEM of 4–8 animals per group. * *p* < 0.05; ** *p* < 0.01 according to *t* test.

The NF-κB signaling pathway is a key mechanism involved in the inflammatory and immune responses. In several peritoneal damage models, the activation of the NF-κB pathway has been reported [26,27]. In mice treated with CHX for 10 days, we found increased levels of the phosphorylated NF-κB subunit p65 (p-NF-κB p65) and phosphorylated levels of the NF-κB inhibitor IκBα (p-IκBα), which indicate the activation of the NF-κB pathway (Figure 3A). The JQ1 treatment significantly reduced the levels of p-IκBα but not the levels of p-NF-κB p65 (Figure 3A). Additionally, we observed that CHX induced genetic expression levels of several inflammatory markers, whereas the JQ1 treatment was able to significantly decrease the expressions of these markers, including genes encoding chemokines, such as C-C motif (*Ccl5*, *Ccl2*, and *Ccl8*) and C-X-C motif (*Cxcl10*) ligands (Figure 3B), together with decreased expressions of genes encoding IL-1β (*Il1b*) and TNF-α (*Tnfa*) cytokines (Figure 3C). These results strongly suggest BET inhibition reduces the peritoneal inflammation induced experimentally in mice by modulating NF-κB/inflammatory gene transcription.

### 3.4. BET Inhibition Modulated Oxidative-Stress-Related Factors in CHX-Treated Mice

**Figure 4:**
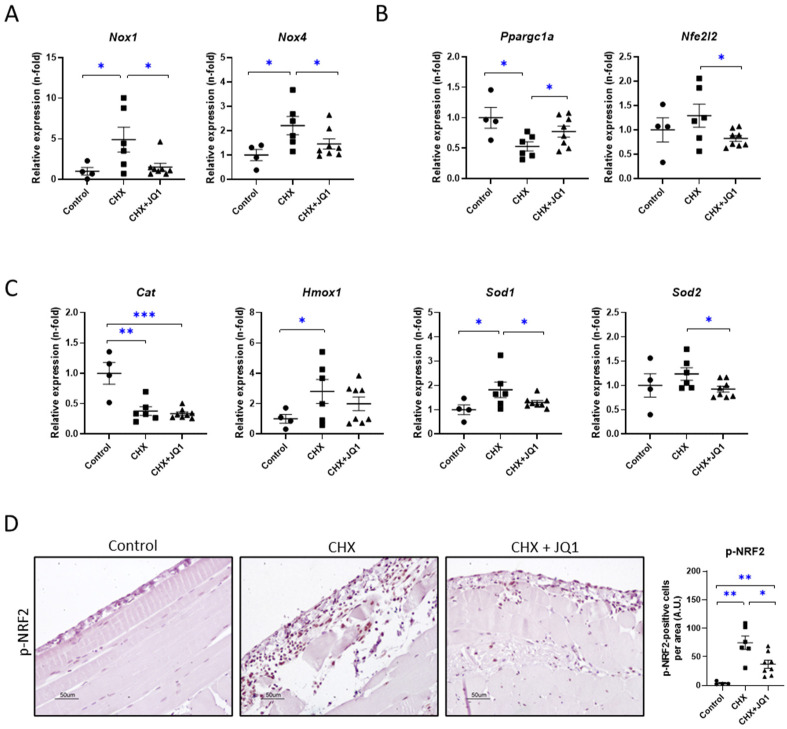
JQ1 restored expression levels of CHX-induced oxidative-stress markers and antioxidant-response enzymes. Relative expressions of genes encoding pro-oxidant enzymes (**A**), oxidation-related transcription factors (**B**), and antioxidant enzymes (**C**) were assayed using RT-qPCR and calculated using the 2^−ΔΔCt^ method, and Gapdh was used as the normalizer gene. (**D**) JQ1 inhibited CHX-induced NRF2 pathway activation in the damaged peritoneum. Phosphorylated NRF2-positive cells were measured using immunohistochemistry and a specific antibody. Microscopy images correspond to a representative animal from each group (200× magnification). Scale bar: 50 μm. Results are expressed as the mean ± SEM (*n* = 4–8 animals per group). * *p* < 0.05; ** *p* < 0.01; *** *p* < 0.001 according to *t* test.

To evaluate whether oxidative stress is induced in this peritoneal damage model, we assayed several oxidation-related factors. We observed that CHX exposure induced the genetic expressions of NAPDH oxidases 1 and 4 (*Nox1* and *Nox4*, respectively), two relevant pro-oxidant enzymes (Figure 4A). Interestingly, the JQ1 treatment restored the *Nox1* and *Nox4* expression levels in the peritonea of the CHX-treated mice. Additionally, we observed a reduction in the genetic expression levels of the master transcriptional coactivator PGC-1α (*Ppargc1a* gene) in the CHX-injured peritoneum, and the JQ1 treatment restored its levels (Figure 4B). PGC-1α is a well-known regulator of the mitochondrial metabolism, including oxidative phosphorylation and reactive oxygen species detoxification [28]. Moreover, the CHX mouse group showed diverse changes in the genetic expressions of antioxidant response enzymes in the peritoneum, such as a decreased expression of catalase (*Cat*) and increased expressions of heme oxygenase 1 (*Hmox1*) and superoxide dismutase 1 (*Sod1*). JQ1 was able to restore the Sod1 genetic expression (Figure 4C). Next, we evaluated the levels of the oxidative-stress regulator nuclear factor erythroid 2-related factor 2 (NRF2). Although the NRF2 coding gene (*Nfe2l2*) did not exhibit changes in its expression levels in response to CHX exposure (Figure 4B), we observed an increased number of p-NRF2-positive cells in the CHX-treated mouse peritoneum, showing the activation of the NRF2 pathway (Figure 4D). Importantly, the JQ1 treatment significantly diminished the number of p-NRF2-positive cells in the damaged peritoneum (Figure 4D), which is associated with the restoration of NRF2-related gene levels. Together, these data indicate that BET inhibition attenuates CHX-induced oxidative stress.

### 3.5. BET Inhibition Diminished Profibrotic- and Proinflammatory-Related Responses in Cultured Mesothelial Cells

Next, we evaluated the in vitro effect of BET inhibition on MeT-5A and effluent-derived primary mesothelial cells. To inhibit BET proteins, the cells were pretreated with JQ1 at a dose of 1 μM for 3 h prior to exposure to different stimuli.

**Figure 5:**
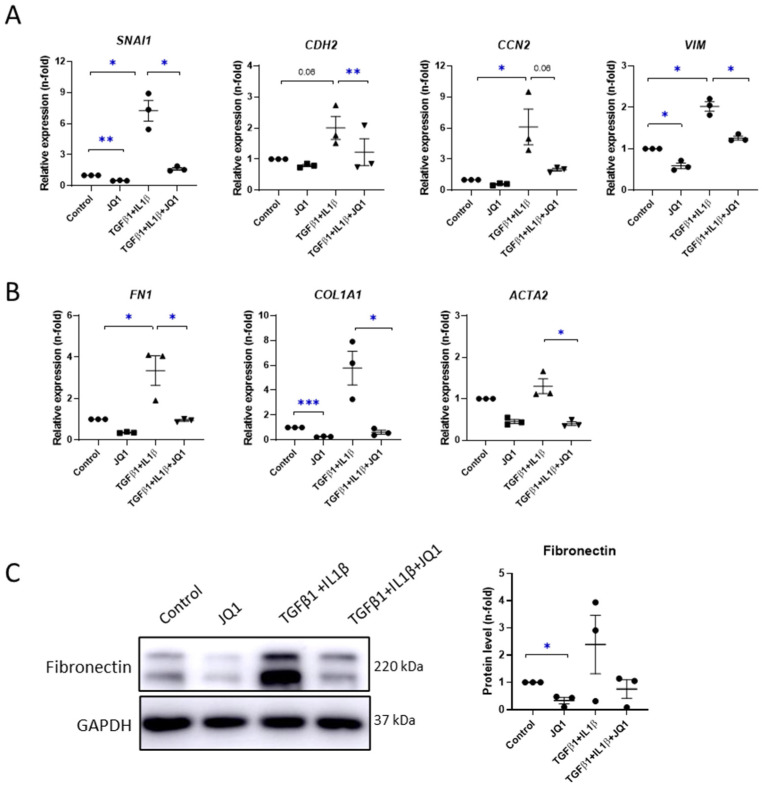
JQ1 prevented TGFβ-induced mesothelial-to-mesenchymal transition (MMT) in vitro. MeT-5A cells were pretreated with JQ1 (1 µM) for 3 h and then stimulated with 2 ng/mL TGF-β1 and 5 ng/mL IL-1β for 24 h. (**A**,**B**) Genetic expressions of *SNAI1*, *CDH2*, *CCN2*, *VIM*, *FN1*, *COL1A1*, and *ACTA2* were assayed using RT-qPCR. *GAPDH* levels were used for normalization. (**C**) Left: representative image of western blot analysis showing the protein expression of fibronectin from total lysates of MeT-5A cells. GAPDH was detected as a loading control. Right: densiometric quantification of western blot images of treated MeT-5A cells compared to control. Results are represented as n-folds with respect to control and are expressed as the mean ± SEM of three independent experiments. * *p* < 0.05; ** *p* < 0.01; *** *p* < 0.001 according to *t* test.

First, we evaluated the effects of JQ1 on peritoneal fibrosis outcomes by stimulating MeT-5A cells with TGF-β1 and IL-1β for 24 h, a combination previously described to induce MMT [4]. We determined the changes in the genetic expression levels of several MMT markers, such as Snail (*SNA1* gene), N-cadherin (*CDH2* gene), connective tissue growth factor (*CCN2* gene), and vimentin (*VIM* gene). We observed that JQ1 downregulated all these genes in TGFβ + IL-1β-stimulated cells (Figure 5A). JQ1 also restored the genetic expression levels of extracellular matrix-remodeling-associated markers, such as fibronectin (*FN1*), collagen type 1 (*COL1A1*), and α-smooth muscle actin (*ACTA2*) (Figure 5B), together with protein levels of fibronectin (Figure 5C).

**Figure 6:**
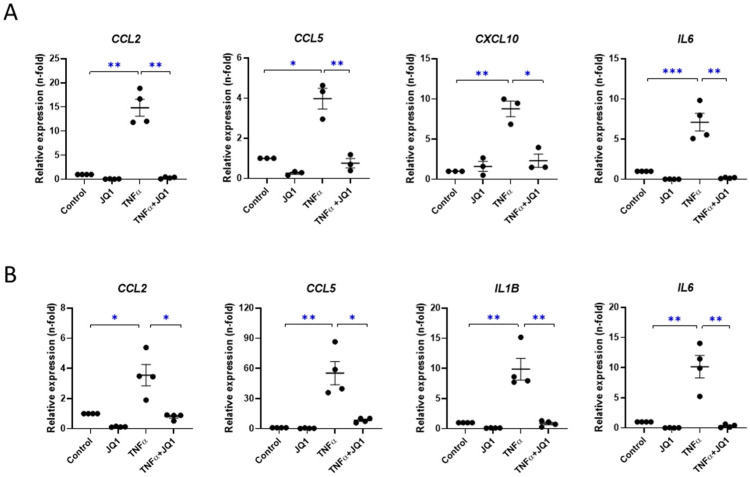
JQ1 strongly reduced TNF-α-dependent inflammatory response in cultured MeT-5A and primary mesothelial cells (MCs). (**A**) MeT-5A cells and (**B**) primary MCs were pretreated with JQ1 (1µM) for 3 h and then stimulated with 5 ng/mL TNF-α for 24 h. Genetic expression levels were evaluated using RT-PCR. *GAPDH* levels were used for normalization. Results are represented as n-folds with respect to control and are expressed as the mean ± SEM of four independent experiments. * *p* < 0.05; ** *p* < 0.01; *** *p* < 0.001 according to *t* test.

Additionally, we provide evidence that JQ1 strongly downmodulated proinflammatory genes induced by TNF-α, such as *CCL2*, *CCL5*, *CXCL10*, *IL1B*, and *IL-6*, both in MeT-5A cells (Figure 6A) and in primary MCs (Figure 6B). All together, these results show that BET inhibition blocked MMT and inflammation in mesothelial cells.

### 3.6. BET Inhibition Reduced Oxidative Stress in Cultured Mesothelial Cells

**Figure 7:**
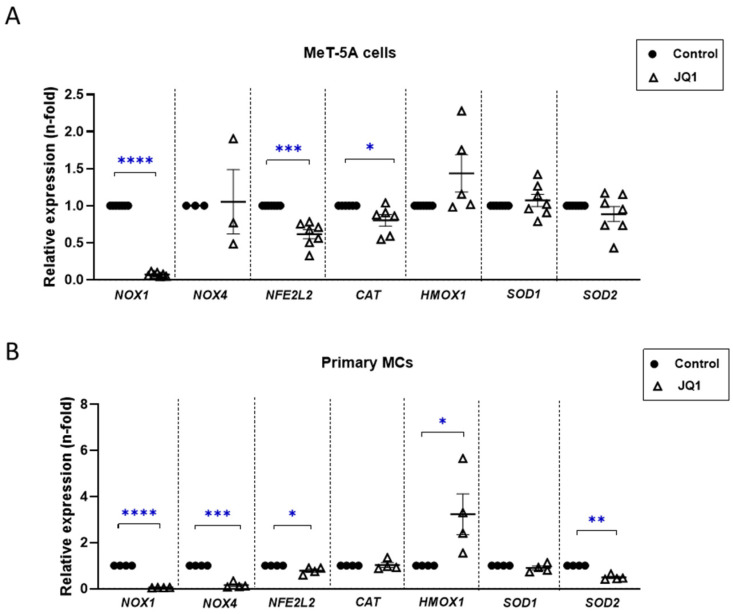
JQ1 regulated oxidative-stress responses in cultured mesothelial cells. MeT-5A (**A**) and Primary MCs (**B**) were treated with 1 µM JQ1 for 24 h and then the total RNA was isolated for genetic expression analyzes using RT-qPCR. Graphs show relative expressions of pro-oxidant enzyme-encoding genes *NOX1* and *NOX4*, NRF-2-encoding gene *NFE2L2*, and its target antioxidant enzymes catalase (*CAT*), heme oxygenase (*HMOX1*), and superoxide dismutases *SOD1* and *SOD2*. *GAPDH* gene levels were used for normalization. Results are represented as n-folds with respect to control and expressed as the mean ± SEM of at least four independent experiments. * *p* < 0.05; ** *p* < 0.01; *** *p* < 0.001; **** *p* < 0.0001 according to *t* test.

Following the results obtained in the in vivo analysis, we analyzed the role of JQ1 in the modulation of the oxidative-stress response in mesothelial cells. For this purpose, MeT-5A cells and primary MCs were treated with JQ1 at a dose of 1 μM for 24 h. In general, we observed that JQ1 downregulated the genetic expression of oxidative markers in both MeT-5A (Figure 7A) and primary MCs (Figure 7B). In particular, BET inhibition significantly decreased the genetic expression levels of pro-oxidant enzymes (*NOX1* and *NOX4*). The gene expression levels of the antioxidant transcription factor NRF2 (*NFE2L2* gene) and its target gene *CAT* (encoding catalase, an antioxidant enzyme) were also downregulated, probably owing to a slight global oxidative stress. On the other hand, anti-oxidative markers, such as *HMOX1* and *SOD1*, either were upregulated or did not change in both mesothelial cell cultures; while the level of *SOD2*, encoding mitochondrial superoxide dismutase, decreased in response to the JQ1 treatment. These data demonstrated that BET inhibition modulates the oxidative-stress response in mesothelial cells.

## 4. Discussion

In the last few years, novel therapeutic agents, including those used in the current clinical treatments of CKD patients, have been assayed in preclinical models of peritoneal damage [3]. In this paper, we describe for the first time the beneficial effects of BET inhibition on an experimental murine model of CHX-induced peritoneal damage. We have found that treatment with JQ1 significantly diminished PM thickening and inflammatory cell infiltration (monocytes/macrophages and T lymphocytes), which was mainly mediated by the inhibition of NF-κB/NRF2 pathways and the modulation of proinflammatory- and redox-controlled genes.

Many experimental data have demonstrated that epigenetic drugs targeting BRDs exert protective effects on proliferative, inflammatory, and fibrotic diseases [29,30,31,32,33]. Different preclinical studies have demonstrated that the anti-inflammatory actions of JQ1 were mediated by the inhibition of the genetic expression of important proinflammatory factors [29,30,31,33]. The mechanism involved in the JQ1 anti-inflammatory action is mediated by the gene transcription inhibition through the blockade of the interaction of bromodomain, present in BET proteins, with acetylated lysine residues in proteins, including histones and transcription factors [29,30,31]. Our results obtained using the mouse model of CHX-induced peritoneal damage showed that the JQ1 treatment diminished the peritoneal gene overexpression of the key chemokines involved in the recruitment and activation of inflammatory cells, including *Ccl2*, *Ccl5*, and *Ccl8*, associated with diminished inflammatory cell infiltration, mainly macrophages and T lymphocytes, in submesothelial areas of the peritoneum. Remarkably, previous in vitro studies have demonstrated that JQ1 directly regulates the genetic expression of these proinflammatory factors by blocking BDR4 binding to acetylated histones and controlling their transcriptional activation [16,33], thus explaining the anti-inflammatory mechanism of BET inhibitors.

The repeated exposure of the PM to PD fluids can induce a local inflammatory response by activating MCs, altering the homeostasis of resident macrophages, and recruiting various inflammatory cells to the submesothelial compact zone [3]. Initial in vitro experiments showed that the stimulation of MCs with inflammatory factors, such as IL-1β, lipopolysaccharides (LPS), or high glucose concentrations, induced the production of several cytokines and chemokines, including IL-6, TNF-α, CCL2, and CCL3, which favored the recruitment and activation of mononuclear cells [34,35]. More recently, peritoneal mesothelium-derived chemokines have been proposed as potential therapeutic targets. The macrophage–mesothelial cell crosstalk through the interaction of fractalkine receptor CX3CR1-CX3CL1 enhances MC-induced TGF-β production, promoting peritoneal fibrosis in response to dialysate exposure [36]. CXCL1 induced in MCs in response to IL-17 exposure displays angiogenic activity and can subsequently control vascular remodeling in dialyzed peritonea [37]. In this study, we have shown that MCs, both from PD patients and cell lines, incubated in a proinflammatory environment, exert a transcriptional reprogramming, including the upregulation of proinflammatory genes, such as IL-6, IL-1β, CCL2, and CCL5, which was prevented by the JQ1 treatment. These findings suggest that the beneficial effects of BET inhibitors may be due to the local inhibition of proinflammatory gene upregulation in activated MCs.

The role of macrophages in the progression of peritoneal injury is well documented. Systemic monocyte/macrophage depletion in mice results in the overall inhibition of peritoneal fibrosis induced by the instillation of PD fluids [38]. In PD patients, several chemokines, such as CCL18, released by activated macrophages owing to peritonitis episodes or long-term PD have been correlated with decreased peritoneal function and may contribute to peritoneal fibrosis [39]. In CHX-induced damage, Ccl8, the mouse functional analog of human CCL18 [40], was the most upregulated chemokine in the peritoneum, as we have observed in this study. Moreover, the functional blockade of CCL8, using a CCR8 inhibitor, prevented PM thickening and macrophage infiltration into the peritoneum [41,42]. In this study, we have found that JQ1 diminished the peritoneal *Ccl8* overexpression. Accordingly, JQ1 modulates macrophage responses, such as the GM-CSF-induced self-renewal and IL-4-induced M2 polarization of peritoneal macrophages, by regulating the activities of c-Myc, KLF4, Mafb, and IRF4 [43]. Recent studies showing increased expressions of the M2 macrophage phenotype markers CD206, TGF-β, Ym-1, Arginase-1, and Ccl17 in mouse models of peritoneal injury [44] have suggested a profibrotic phenotype that promotes fibroblast activation [44]. Our data showing a lowered macrophage infiltration and the synthesis of related cytokines, such as *Ccl2* and *Ccl8*, could also explain the reduction in the PM thickness and fibrosis induced by the BET inhibition.

One important mechanism involved in the anti-inflammatory properties of JQ1 is the inhibition of the NF-κB inflammatory pathway. In resting cells, NF-κB remains inactive in the cytosol, and exposure to proinflammatory factors can lead to the activation of the canonical NF-κB pathway [45]. The modulation of the transcriptional activity of p65/NF-κB by Ser536 phosphorylation is involved in the upregulation of many proinflammatory genes, including IL-6 and CCL5 [46]. BRD4 binds to acetylated residues in NF-κB and avoids RelA/p65 NF-κB ubiquitination and its subsequent degradation, thus keeping transcription active [46]. JQ1 can bind to the acetylated Ser310 of p65/NF-κB, leading to the release of the p65 subunit from the active NF-κB complex in the nuclei and subsequent p65 ubiquitination and degradation. By this mechanism, JQ1 inhibited NF-κB-downstream proinflammatory genes [33,47]. The NF-κB signaling pathway is activated in response to damage to the peritoneum. The exposure of cultured human peritoneal MCs to PD fluids activated the NF-κB pathway and subsequent TNFR1 signaling [48]. Very recent studies have found that the inhibition of NF-κB, using the natural compound parthenolide, improved the peritoneal damage induced by PDF exposure by locally targeting NF-κB-regulated proinflammatory cytokines, such as IL-6, TNF-α, and CCL5 [49]. Although peritoneal damage in this study was not induced by conventional glucose-based PDF, we have also activated the NF-κB pathway by repeated exposure of the mouse model to the toxin CHX, which validates the use of this model for anti-inflammatory drug testing. Previous studies have found that BET inhibition with JQ1 reduces proinflammatory responses under the transcriptional control of NF-κB in human renal tubular cells and in mouse models of renal damage [33]. We have described in this study that in CHX-induced peritoneal damage, JQ1 inhibited NF-κB activation, confirming this anti-inflammatory mechanism in experimentally induced peritoneal damage and extending the beneficial effects of iBET inhibitors to preserve the PM integrity.

The peritoneum is composed of a monolayer of MCs with an epithelial-like cobblestone shape covering the continuum of the peritoneal cavity. Upon the chronic exposure of MCs to PD fluids, these cells can undergo a process called MMT in which they lose the epithelial characteristics and acquire mesenchymal features, as well as mobility and tissue invasion [50]. The role of MMT in peritoneal fibrosis has been well established Besides downregulating the genes involved in the immune response, BET inhibition also dampens the cellular activation, proliferation, apoptosis programs, and fibrotic process [51,52,53,54,55]. Our experiments on cultured MeT-5A MCs stimulated under profibrotic conditions showed that the JQ1 treatment could restore the changes in the expressions of mesenchymal phenotype-associated genes, including snail, vimentin, and profibrotic factors, supporting the role of BET inhibition in mediating MMT reversal. Accordingly, JQ1 restored the phenotypic changes and profibrotic factor upregulation in other cell types, including TGF-β1-activated tubular epithelial cells [56,57,58,59], or in mechanical-injury-induced corneal scarring [60]. In renal cell carcinoma, the BRD4 knockdown and JQ1 treatment suppressed the epithelial-to-mesenchymal transition (EMT) and tumor growth progression via caspase-1-dependent pyroptosis in vitro and in vivo [61]. Moreover, in the CHX-induced model of peritoneal damage, we have found that JQ1 significantly diminished PM thickening and fibrosis, reversed changes in MMT markers, and inhibited SMAD2/3 activation. Previous studies have found that epigenetic drugs targeting histone acetylation/deacetylation (HAT or HDAC inhibitors), histone/DNA methylation, or histone methyltransferase activities, modulated experimentally induced peritoneal fibrosis [6,7,8,62]. In addition, some of these epigenetic drugs also prevented MMT changes [9,10,24,63]. Our findings showing MMT phenotypic restoration in cultured MCs and in the CHX-damaged peritoneum support the hypothesis that epigenetic drugs targeting BET proteins could also exert antifibrotic effects on peritoneal fibrosis by modulating phenotypic changes.

Reactive oxygen species (ROS) are fundamental mediators for numerous cellular processes in homeostasis, such as growth, survival, and proliferation [64]. Oxidative stress is connected with inflammation and mitochondrial metabolism by several intracellular signaling systems, including NF-κB [65] and PGC-1α [28]. In peritoneal damage, ROS are involved in many of those processes, including inflammation, MMT, and senescence [65,66,67,68], some of which target PGC-1α [69] and modulate oxidative phosphorylation or fatty-acid metabolism-related proteins [70]. In human peritoneal MCs, exposure to high-glucose-concentration PD fluids induced excess ROS production, lipid peroxidation, and oxidative DNA damage [71]. Recent studies have demonstrated that BET inhibition significantly suppressed oxidative stress, as described in studies on experimental models of kidney damage [55,72,73,74]. The earliest studies on cultured cells showed that JQ1 prevents H_2_O_2_-induced intracellular ROS production in rat chondrocyte cells [75]. In cultured tubular epithelial cells, JQ1 prevented TGF-β1-induced oxidative stress, as evaluated based on a mitoSOX^™^ assay and the genetic expression levels of catalase [74]. Mechanistically, JQ1 induced the expressions of antioxidative genes (Keap1/Nrf2 and Hmox1) while decreasing the expression of pro-oxidative factors, such as NOS, thus decreasing the production of NO species [76,77,78], which prevented mitochondrial ROS production [74,79], suppressed NOX4 activity [56,80], and modulated transcription factor activities, such as FOXO4 [73]. In our model of CHX-induced peritoneal damage, oxidative stress was also diminished by JQ1, as shown by the downregulation of the NOX1, NOX4, and PGC-1α gene levels.

NRF2 is a transcription factor that participates in the protective response against oxidative stress and in the modulation of the inflammatory response [81]. An in vitro model, using peritoneal MCs derived from diabetic rats exposed to PD solutions, showed an increase in oxidative stress, as determined by elevated ROS production and NRF2 expression [82]. Some indirect evidence supports the hypothesis that the modulation of the NRF2/redox pathway can protect against PM damage, as proposed in a study using asiaticoside, a component of triterpenoid saponins, which inhibited TGF-β1-induced MMT [83]. Interestingly, CKD patients in PD therapy presented elevated plasmatic concentrations of malondialdehyde and upregulated gene and protein expressions of NRF2 and SOD2 compared to healthy subjects, suggesting the important contribution of the NRF2/oxidative-stress pathway in peritoneal damage [84]. We have described in this study that CHX-induced peritoneal damage activated the NRF2 pathway, as shown by the nuclear expression of p-NRF2 in cells located in the submesothelial layer. Moreover, the treatment of mice with JQ1 diminished the NRF2 pathway activation in the damaged peritoneum, which is associated with the modulation of the antioxidant response to NRF2-downstream targets, such as HMOX1 and SOD2. Accordingly, BRD4 inhibition regulates the oxidative response, diminishing the expressions of NRF2 and related genes, such as HMOX1 and KEAP1, in activated HEK293T cells [76]. On the contrary, in primary human airway smooth muscle cells and human monocytic cells, JQ1 induced the activation of NRF2-dependent transcription, including the expressions of HMOX1 and NADPH quinone oxidoreductase 1 [85]. In cisplatin-induced nephrotoxicity in mice, the JQ1 treatment increased the genetic expressions of NRF2 and HMOX1 [77]. These data suggest that the NRF2 pathway activation varies between cells and pathological conditions.

In clinical practice, a small number of epigenetic inhibitors have been approved by regulatory authorities so far [86]. Regarding BET inhibitors in particular, their effects have mainly been tested on leukemias and solid tumors [87]. In the treatment of chronic inflammatory diseases, it is believed that side effects and incomplete knowledge of multiple molecular mechanisms elicited by epidrugs have prevented more widespread analysis in clinical trials. To date, only a few clinical trials have been performed for non-cancerous diseases. In patients with cardiovascular diseases, the BET inhibitor apabetalone has shown beneficial effects on atherosclerosis (NCT01067820) and acute coronary syndrome (NCT02586155). In Fabry kidney disease, there was a phase II study that evaluated the effects of apabetalone treatment on several outcomes, including cardiovascular damage markers, but the study was withdrawn owing to changes in developmental priorities (NCT03228940). Thus, according to the available preclinical evidence, clinical trials using assays to determine the effects of BET inhibitors on kidney diseases and related complications may be feasible.

## 5. Conclusions

**Figure 8:**
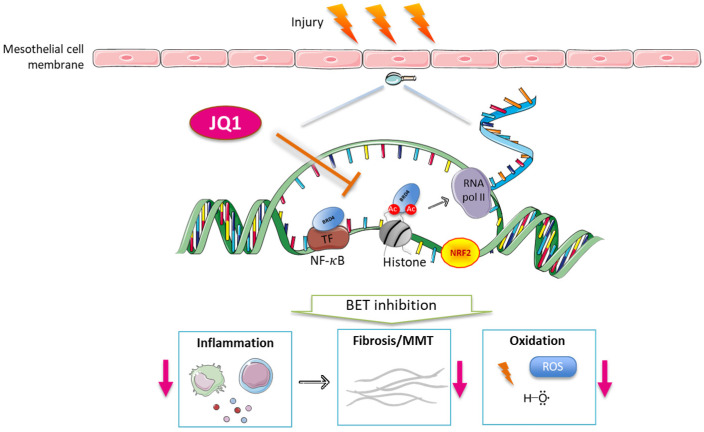
Mechanisms involved in the beneficial effects of JQ1 on peritoneal damage. BRD4 is a BET protein that binds to acetylated histones and transcription factors, such as NF-κB, and stimulates RNA pol II-dependent transcription. JQ1, by inhibiting BRD4 actions, decreases damage to peritoneum-derived mesothelial cells through reducing inflammation, mesothelial-to-mesenchymal transition (MMT), and fibrosis and downmodulating oxidative responses. Red arrows indicate reduction on biological processes.

A large body of preclinical evidence supports the beneficial effects of BET inhibitors on chronic inflammatory disorders, including kidney diseases and the associated complications observed in ESKD patients. The data presented in this study have revealed that the blockage of BET proteins with the small-molecule inhibitor JQ1 demonstrated beneficial effects on peritoneal damage by targeting key molecular pathways, including NF-κB, downstream proinflammatory genes, and NRF2/redox-controlled genes (Figure 8). These findings may offer a pharmacological alternative for the treatment of peritoneal inflammation and fibrosis outcomes during PD replacement therapy. Although no clinical trial has been conducted on PD patients, a phase II study on apabetalone in patients with ESKD treated with hemodialysis is currently under recruitment (NCT03160430). In this regard, our preclinical results support the development of future clinical trials on ESKD patients undergoing PD to evaluate not only renal and cardiovascular outcomes but also the peritoneal membrane function.

## Data Availability

Data is contained within the article and supplementary material.

## Supplementary Materials

The following supporting information can be downloaded at https://www.mdpi.com/article/10.3390/antiox12122055/s1, Supplementary Table S1: Demograhic and clinical features of PD patients; Supplementary Table S2: Pre-designed assays used for qPCR.
