# Supplementary material for: BET Protein Inhibitor JQ1 Ameliorates Experimental Peritoneal Damage by Inhibition of Inflammation and Oxidative Stress
Source: Antioxidants (Basel). 2023 Nov 29;12(12):2055. doi: 10.3390/antiox12122055 (PMC10740563; doi:10.3390/antiox12122055)
Supplement: Supplementary file 1 [file antioxidants-12-02055-s001.zip › antioxidants-2701850-supplementary.pdf]

Supplementary Table S1. **Demograhic and clinical features of PD patients**.

Patient	Sex	Age (years)	Cause of kidney failure	Diabetes	Hypertension	Months on PD	PD technique	Exchanges	Glucose (mg/dL)	Peritonitis	Hemoperitoneum	Escapes
1	M	57	T1DM	Yes	Yes	56	CCPD	20 L/24 h	1815	No	No	No
2	M	77	Unknown	No	Yes	5	CAPD	1	1360	No	No	No
3	F	65	ADPKD	No	Yes	11	CAPD	1	2270	No	No	No
4	F	29	Extracapillary FGN	No	Yes	3	CAPD	1	2270	No	No	No

M: male; F: female; T1DM; type 1 diabetes mellitus; ADPKD: autosomal dominant polycystic kidney disease; FGN: fibrillary glomerulonephritis; CCPD: Continuous cyclic peritoneal dialysis; CAPD: Continuous ambulatory peritoneal dialysis.

Supplementary Table S2. Pre-designed assays used for qPCR.

**Specie**	**Gene**	**Assay ID**	**Company**
Mouse	*Ccl2*	Mm.PT.58.42151692	Integrated DNA Technologies
Mouse	*Ccl5*	Mm.PT.58.43548565	Integrated DNA Technologies
Mouse	*Ccl8*	Mm01297183_m1	Applied Biosystems
Mouse	*Cxcl10*	Mm.PT.58.43548565	Integrated DNA Technologies
Mouse	*Il1b*	Mm.PT.58.41616450	Integrated DNA Technologies
Mouse	*Tnfa*	Mm00443258_m1	Applied Biosystems
Mouse	*Snai1*	Mm00441533_g1	Applied Biosystems
Mouse	*Cdh2*	Mm.PT.58.12378183	Integrated DNA Technologies
Mouse	*Acta2*	Mm.PT.58.16320644	Integrated DNA Technologies
Mouse	*Nox1*	Mm.PT.58.29694286	Integrated DNA Technologies
Mouse	*Nox4*	Mm.PT.58.8820983	Integrated DNA Technologies
Mouse	*Ppargc1a*	Mm.PT.58.16192665	Integrated DNA Technologies
Mouse	*Nfe2l2*	Mm.PT.58.29108649	Integrated DNA Technologies
Mouse	*Cat*	Mm.PT.58.12825133	Integrated DNA Technologies
Mouse	*Hmox1*	Mm.PT.58.8600055	Integrated DNA Technologies
Mouse	*Sod1*	Mm.PT.58.12368303	Integrated DNA Technologies
Mouse	*Sod2*	Mm.PT.58.14276358	Integrated DNA Technologies
Mouse	*Gapdh*	Mm99999915_g1	Applied Biosystems
Human	*CCL2*	Hs.PT.58.45467977	Integrated DNA Technologies
Human	*CCL5*	Hs.PT.58.1724551	Integrated DNA Technologies
Human	*IL1B*	Hs.PT.58.1518186	Integrated DNA Technologies
Human	*IL6*	Hs.PT.58.40226675	Integrated DNA Technologies
Human	*SNAI1*	Hs.PT.58.2984401	Integrated DNA Technologies
Human	*CDH2*	Hs.PT.58.26024443	Integrated DNA Technologies
Human	*CCN2*	Hs.PT.58.14485164.g	Integrated DNA Technologies
Human	*VIM*	Hs00185584_g1	Applied Biosystems
Human	*FN1*	Hs.PT.58.40005963	Integrated DNA Technologies
Human	*COL1A1*	Hs.PT.58.15517795	Integrated DNA Technologies
Human	*ACTA2*	Hs.PT.56a.2542642	Integrated DNA Technologies
Human	*NOX1*	Hs.PT.58.381842	Integrated DNA Technologies
Human	*NOX4*	Hs.PT.58.3866448	Integrated DNA Technologies
Human	*NFE2L2*	Hs.PT.58.28159373	Integrated DNA Technologies
Human	*CAT*	Hs.PT.56a.25069031	Integrated DNA Technologies
Human	*HMOX1*	Hs.PT.58.45340055	Integrated DNA Technologies
Human	*SOD1*	Hs.PT.58.20593019	Integrated DNA Technologies
Human	*SOD2*	Hs.PT.58.25533008	Integrated DNA Technologies
Human	*GAPDH*	Hs02786624_g1	Applied Biosystems
